# Clinical Effect of Photobiomodulation on Wound Healing of Diabetic Foot Ulcers: Does Skin Color Needs to Be Considered?

**DOI:** 10.1155/2022/3312840

**Published:** 2022-12-17

**Authors:** Thabo Dhlamini, Nicolette Nadene Houreld

**Affiliations:** Laser Research Centre, Faculty of Health Sciences, University of Johannesburg, P.O. Box 17011, Doornfontein, South Africa 2028

## Abstract

Diabetic foot ulcers (DFUs) are one of the most common complications of diabetes. DFUs impede patients' quality of life and are known to be unresponsive to conventional therapy. Photobiomodulation (PBM) is a pain-free, noninvasive treatment method that has been shown to promote chronic wound healing and has been successfully used for the treatment of DFUs. Since skin tone and color can affect the way light interacts with tissue, studies should take this into consideration when determining protocols for the use of PBM. This review is aimed at critically evaluating data of existing studies conducted to evaluate the clinical effect of PBM on DFUs, taking skin color into consideration. A literature search was conducted and resulted in articles on cell studies, animal studies, and clinical trials. Only 13 clinical trials and 2 clinical case studies were adopted and used in this review. All the clinical trials adopted for this review show evidence that PBM together with conventional treatment results in an increased healing rate of DFUs; however, only one study adjusted their protocol according to skin color. There are not enough studies conducted on people of color to determine the safety and efficacy of PBM therapy in such ethnic groups. Future randomized, placebo-controlled clinical trials are necessary on PBM and DFUs and should take skin color into consideration.

## 1. Introduction

### 1.1. Diabetes Mellitus (DM)

DM is a universal widespread metabolic disease in which a person has high-blood glucose. There are two types of DM: type I, known as “insulin independent diabetes”, which results from the body's inability to produce and secrete insulin and type II, known as “non-insulin-dependent diabetes”, which results from the inability of the cells to respond to the insulin produced by the body [1]. According to the International Diabetes Federation (IDF), there are 536.6 million adults aged 20-79 years living with DM globally (24 million in Africa), a figure which is predicted to increase to 696 million (10.2% of the population) by 2030, and 783.2 million (12.2%) by 2045 [2]. Globally, DM is among the top 10 causes of death [2]. People living with DM are at increased risk of developing various life-threatening complications, including chronic diabetic foot ulcers (DFUs), leading to an increased need for medical care, a reduced quality of life, and undue financial and psychological stress.

### 1.2. Diabetic Foot Ulcers (DFUs)

People with DM are subjected to foot complications that develop due to long periods of hyperglycaemia. Diabetic foot neuropathy and peripheral vascular disease are two of the main causes of severe diabetic foot complications. The concurrent occurrence of diabetic foot complications leads to ulcerations [3]. The highly causative factor of DFUs is peripheral neuropathy (sensory, motor, and autonomic). Sensory neuropathy leads to patients experiencing a loss of protective sensation which results in susceptibility to physical and thermal trauma, and thus increased risk of ulceration [4]. Plantar pressure and friction that result from limited joint mobility and foot deformity caused by diabetes also causes foot ulcerations. This friction and increased pressure cause breakdown of the skin and lead to increased risk of infections, blister formation, callus formation, and ulcerations, and in severe cases, necessitates amputation [5]. Diabetic foot and lower limb complications affect 40 to 60 million people globally with DM and it is the principal cause of morbidity in diabetic patients [6]. It is estimated that the lifetime risk for developing a DFU is as high as 25%, with a global prevalence of 6.3% [7, 8].

### 1.3. Classification of Diabetic Foot Ulcers (DFUs)

Taking size and depth into consideration, DFUs can be classified as mild (superficial), moderate (deeper), and severe (systemic signs). However, in 1976, Meggitt-Wagner designed a classification system for DFUs, and this classification has been used over the years to date. This classification system is based on wound depth and tissue viability. There are six classifications from this system and ulcers are graded on a scale from 0 to 5. Grade 0 describes a preulcerative or postulcerative site; grade 1 describes a superficial ulcer; grade 2 describes ulcers which penetrate the tendon or joint capsule; grade 3 describes ulcers which involve deeper tissues; grade 4 describes ulcers that involve gangrene of the forefoot; and grade 5 includes whole foot gangrene involving more than two thirds of the foot [9].

### 1.4. Diabetic Ulceration Management

The management and care of DFUs pose an increasing socioeconomic burden owing to repeated hospital admissions, lengthy treatment plans, and the risk of amputations, incurring extensive healthcare costs [8]. A conventional holistic treatment approach is used to treat DFUs involving an interdisciplinary team of healthcare professionals, as well as the patients and caregivers. This treatment approach includes debridement, cleaning and dressing of the wound, and in some cases, antibiotic therapy and pain management [10]. Debridement involves the removal of dead, necrotic tissue, or foreign material from and around a wound to expose healthy tissue and is part of the standard of care for DFUs [11]. There are different methods of debridement, including sharp/surgical, biological, autolytic, biochemical, and osmotic debridement. Wound dressings are applied to the wounds to clean, cover, and protect the wound from the external environment, to protect the wound from infection, and to promote healing. Wound dressings should provide a moist environment as well as remove excess exudate. There are different types of wound dressings each with their own unique properties available for the management of DFUs, including hydrogels, hydrocolloids, alginates, foam, silver-induced dressings, growth factor-impregnated dressings, and silicon-impregnated atraumatic dressings, and each dressing is suitable for different wound conditions [12]. Part of the treatment approach for DFUs also includes offloading for pressure redistribution and relief in pressure areas of the foot. Offloading results in tissue trauma prevention and facilitation of wound healing [13].

Despite having all these treatments in place, only a 50% healing rate is achieved, and the global risk for recurrence stands at 50% [8, 14]. There is a need for the development of alternative, safe, efficient treatments for DFUs. A new adjunct treatment known as photobiomodulation (PBM) for DFUs has emerged and involves the use of low-powered light at specific wavelengths. With efficient intervention and treatment of DFUs, lower limb amputations can be avoided, thus reducing the socioeconomic burden and improving the quality of life of patients.

### 1.5. Photobiomodulation (PBM)

PBM, formally referred to as low-level laser therapy, involves the use of light, typically from lasers or light emitting diodes (LEDs), usually within the visible red and/or near infrared (NIR) electromagnetic spectrum to stimulate healing. PBM is a therapeutic, noninvasive modality that aids in the relief of pain and the reduction of inflammation [15]. PBM has been shown to positively affect the three principal phases of healing, namely, inflammation, proliferation, and maturation in both acute and chronic wounds [16, 17]. A systematic review of PBM for DFUs reported that the therapy has significant potential to become a portable, minimally invasive, easy-to-use, and cost-effective treatment modality for DFUs [18].

Research suggests that the effects of PBM are photochemical and photobiological rather than thermal [19]. The physical properties of skin, including chemical, optical, mechanical, and the concentration, and distribution of components that makes up the tissue (such as melanin) affect the interaction of light with tissue [20]. When light comes into contact with skin, there are four possible outcomes, namely, reflection, scattering, absorption, and transmission [21]. During reflection, approximately 30% of the photons bounce off the skin, and there is no light-tissue interaction [22]. Some of the light that does pass through gets scattered within the tissue; longer wavelengths have a deeper penetration while shorter wavelengths are scattered more [23]. During absorption, the photons are absorbed by chromophores in the tissue. During transmission, the light passes straight through the tissue unhindered, without interacting with the tissue. Both absorption and scattering are dependent on wavelength. Wavelengths < 600 nm are absorbed mainly by melanin and hemoglobin, and wavelengths > 1,200 nm are absorbed by water [21, 24]. There is a so-called “optical window” which refers to the optimum wavelengths to use in PBM and where effective tissue penetration and tissue-light interaction are maximized. This lies between 600–1,070 nm. Wavelengths between 600–700 nm are used to treat superficial tissue as their penetration depth is less, and longer wavelengths between 780–950 nm are used to treat deeper tissues as they penetrate further into tissue [21].

One of the proposed mechanisms of action involves the absorption of photons by mitochondrial cytochrome c oxidase (COX). This enzyme forms part of the mitochondrial electron transport chain and transports electrons from cytochrome c to molecular oxygen. It has been shown that PBM at 660 nm increases the activity of cytochrome c oxidase in irradiated isolated mitochondria, leading to an increase in adenosine triphosphate (ATP) [25]. There is also an increase in intracellular reactive oxygen species (ROS), which is an important messenger for cell signalling pathways and gene transcription. Another theory explains how inhibitory nitric oxide (NO) binds to COX and is photodissociated, freeing the copper and heme centers of COX which can then bind to oxygen [26].

During PBM, the light (photon) energy must be absorbed by the skin and reach subcutaneous tissue. Absorption of this photon energy may be affected by skin color [19], and treatment protocols may need to be adjusted accordingly. The study conducted by Joensen et al., [19] on the thermal effect of a class 3B laser (200 mW; 810 nm) on different skin types within the therapeutic dose showed a consistent thermal effect of laser irradiation, which was strictly limited to the area of irradiation, and that the heat induced was strongly related to skin color. On the contrary, Sithole [27] found no heating effect in diabetic patients with Fitzpatrick skin tones III and V treated with a 1,200 mW LED device. Participants with Fitzpatrick skin tone V felt a mild to moderate change in heat, with no adverse events (blistering or depigmentation). However, changes in skin temperature were not measured in this study. Most studies conducted on PBM for diabetic wound healing have not taken skin color into consideration, and little is known about the efficacy of PBM in individuals with darker skin tones. This leads to the question of whether protocols need to be adjusted according to the different skin tones.

### 1.6. Skin Types

There are several different ethnic groups in South Africa, and they all present with different skin colors. In 1975, Fitzpatrick developed a skin-typing system based on the history of an individual's tendency to tan following exposure to ultraviolet radiation (UVR). There are six classifications from this system, based on the skin color. Type I describes a pale white skin; type II describes white skin; type III describes light brown skin; type IV describes moderate brown skin; type V describes dark brown skin; and type VI describes deeply pigmented dark brown to black skin [28]. This review is aimed at evaluating data of studies conducted to evaluate the clinical effect of PBM on DFUs, taking skin color into consideration.

## 2. Methodology

A review of relevant literature was conducted by database research. The databases include MEDLINE, Google Scholar, Springer, ScienceDirect, and Scopus. These databases were searched for relevant articles published between 2006 and 2021 on the clinical effect of PBM on wound healing of DFUs. The following keyword search strategy was used: “Photobiomodulation AND Diabetes mellitus,” “Photobiomodulation AND diabetic wound healing,” “Photobiomodulation AND diabetic ulcers,” “low level laser therapy AND Diabetes mellitus,” “Effects of LLLT on diabetic wound,” “low level laser therapy AND diabetic wound healing,” “Chronic diabetic wounds,” and “low level laser therapy AND diabetic ulcers”. Only data from articles written in English were included. Articles not written in English were excluded. The search resulted in cell studies, animal studies, and human studies. Only thirteen clinical trials and two clinical case studies were adopted and used in this review. This article was not intended to be a systematic review or meta-analysis but as a “narrative overview”.

## 3. Results and Discussion

Thirteen clinical trials and two clinical case studies were included, and a summary of the studies can be seen in Table 1. The clinical studies all show a similar pattern when it comes to healing of diabetic ulcers or general wounds with PBM. Kazemi-Khoo [29] conducted a clinical case study on the treatment of DFUs with PBM. This study included type 2 diabetic patients (four males with a mean age of 63 years and three females with a mean age of 61 years) with Braden Scale type II and III diabetic ulcers that presented with an average of 6.5 months. The PBM intervention included PBM of the ulcer bed with red light (wavelength 660 nm) and wound margins with infrared light (wavelength 980 nm). There was also intravenous laser irradiation with red light (wavelength 655 nm) and laser acupuncture with an infrared diode laser (wavelength not provided) for LI-11, LI-4, SP-6, PC-6, ST-36, and GB-34 points. Irradiation took place every other day for 10 to 15 sessions (route 1) then continued twice weekly till there was complete recovery (route 2). Complete wound closure took an average of 19 sessions: route 1 with an average of 15 sessions and route 2 with an average of 10 sessions. All cases achieved complete recovery after approximately six months. No side effects were reported by patients, and there was no ulcer relapse at the 6-month follow-up [29]. The author did not report on the skin color nor race of participants; however, based on the images used in the article, we can assume that the participants were of a lighter skin color. Limiting factors of this study included limited information on methodology and contradictory information on the laser parameters in the abstract and methodology section of the manuscript (abstract indicates intravenous PBM was done at a wavelength of 650 nm and power of 1.5 mW, while the methodology section indicates 655 nm with 2 mW). Also, no controls were included.

Saltmarche [30] conducted a prospective comparative clinical trial aimed at testing the effectiveness of PBM in wound healing combined with the Extendicare Wound Prevention and Management Program, a program consisting of a multidisciplinary team of clinicians with an interest and expertise in wounds. The study comprised of 21 open wounds and four “at risk” closed areas. The study was not conducted exclusively on DFUs, and also included pressure wounds and venous wounds. Fifty-seven percent of the wounds were chronic (present for more than three months) and 42.9% of the wounds were acute (present for less than three months). Saltmarche [30] used a laser cluster (wavelength 785 nm). In the first week, the intervention was performed daily for five days, and thereafter, three times weekly up to week 9 or until complete wound closure was observed. The results showed that 61.9% of the open wounds treated with PBM improved and attained >50% wound closure. Nine of the wounds (42.8%) achieved 100% closure and 14.3% of the wounds indicated some improvement, with 23.8% showing no change. This clinical trial showed that there were no differences in healing times between the chronic and the acute wounds. No PBM adverse side effects were noted [30]. There was no reporting on participant skin color or race; however, it was mentioned that for patients with dark skin the dose around the wound periphery was increased by 50%, as melanin absorbs photons and less energy is delivered to the underlying tissue. Limiting factors of this study include the way the results were presented. The researcher did not separate and report on the results of the various ulcer types. These ulcers have different etiologies and could respond differently to PBM. Nonetheless, the author did take skin color into consideration and adjusted the protocol accordingly.

A double-blinded, randomized, placebo-controlled trial was conducted by Minatel et al. [31]. This study tested the hypothesis that combined 660 nm and 890 nm PBM using superluminous diodes would promote healing of diabetic leg ulcers that did not respond to other treatment modalities. The study was carried out on 14 patients with a total of 23 diabetic leg ulcers. Group one had 10 ulcers and group two had 13 ulcers. All patients received conventional therapy twice a week. Group one received conventional therapy and placebo phototherapy (<1.0 J/cm^2^). Group two received conventional therapy together with PBM at 660 nm. Ulcer size was measured on days 0, 15, 30, 45, 60, 75, and 90. The results showed that the mean ulcer healing and granulation rates were higher in group two than in group one. At 30 days, group two had 56% more granulation tissue and a 72% faster healing rate, while the ulcers in group one worsened. By day 90, 58.3% of group two had fully healed ulcers, while only one ulcer was fully healed in the placebo group. Overall, 75% of ulcers in group two achieved 90–100% healing, while none of the ulcers in group two (with the exception of one ulcer) attained >90% healing. No side effects were experienced by the patients in this study [31]. Race nor skin color of the participants was indicated. Other limiting factors for this study included a small sample size and the fact that ulcers in the placebo group were exposed to some red light (660 nm) which could not be avoided due to the blinding nature of the study.

In a randomized clinical trial, Kaviani et al. [32] explored the effect of PBM on chronic diabetic foot wound healing. This study included 23 patients with Wagner grade I and II DFUs of an average of 10 months. In this double-blinded, randomized clinical trial, 10 patients were treated with placebo and 13 patients were treated with PBM at 685 nm. Patients were simultaneously treated with conventional therapy. This trial was set to take place over 20 weeks or until there was complete wound closure. Kaviani et al. [32] noted that the size of the ulcers in the PBM group had significantly decreased at week 4. At week 20, it was noted that 8 of 13 ulcers (66.6%) had complete wound closure in the PBM group, and only 3 of 9 ulcers (38.4%) had complete wound healing in the placebo group. This was not significantly higher than the placebo group, despite the mean time of complete healing being 11 weeks in the PBM group compared to 14 weeks in the placebo group. During the course of the study, two patients from the placebo group were hospitalized and underwent limb amputation, while one patient from the PBM group was hospitalized for infection treatment (no amputation required) [32]. This study provides evidence that PBM can accelerate wound healing in DFUs and shortens the time needed to achieve complete wound healing. Limiting factors include not reporting on the skin color/race of participants; however, the images used indicate that lighter skinned patients participated in this trial, and a small sample number.

Kajagar et al. [33] conducted a study similar to Kaviani et al. [32] whereby they evaluated the efficacy of PBM using a multidiode cluster probe (wavelengths not provided) on wound healing in patients with chronic DFUs. This was a randomized-controlled clinical trial that involved 68 patients with type 2 DM who presented with Wagner grade I foot ulcers for an average of 4 weeks. Patients were randomized into two groups of 34 each. The first group was treated with PBM and conventional therapy, and the second group (control) was only treated with conventional therapy. Kajagar et al. [33] noted that there was significant wound reduction in group one, which received both PBM and conventional therapy, as compared to group two, which only received conventional therapy. The mean reduction in ulcer size was 1043.20 ± 266.62 mm in the PBM-treated group compared to 322.44 ± 85.84 mm in the control group, which was clinically significant. The researchers did not mention participant skin color/race, though from a photograph provided in the article, some participants may have been skin type III or IV; however, this is based on assumptions and not facts provided for in the article.

Feitosa et al. [34] conducted a clinical trial aimed at evaluating the effects of PBM on diabetic ulcers. This was a randomized-controlled clinical study that involved 16 patients with type 2 DM. Eight patients were included in the control group and the other eight were exposed to PBM (wavelength 632.8 nm). This study was designed to detect pain and ulcer size before and after PBM on day 30 of follow-up. At the end of 30 days, the control group displayed no significant decrease in wound size; the patients still experienced pain and one patient required amputation. The average wound area (cm^2^) before treatments in the control group was 2.55 ± 0.77, which increased to 8.43 ± 1.84 after 30 days. In the study group, a significant decrease in wound size was noted and patients reported an improvement in pain. The average wound area (cm^2^) before treatments in the PBM group was 7.98 ± 2.06, which decreased to 2.39 ± 1.26 after 30 days [34]. Skin color nor race of participants was mentioned in this study. Photographs supplied in the article suggest skin types III and less; however, this is again based on assumptions.

Ortíz et al. [35] conducted a randomized-controlled clinical trial on twenty-eight participants comparing the effects of PBM (9 participants), high-voltage-pulsed current (HVPC; 9 participants) and standard wound care (10 participants) on the healing of DFUs localized on the distal leg or feet. Ulcers classified as category I or II according to the Wagner classification system were included. Ulcers were treated for 16 weeks, and DFUs in the control group were treated daily with conventional treatment. The HVPC participants were treated with an electrical simulator and standard wound care. The PBM group was treated with a semiconductor laser diode at 685 nm and standard care. The results showed that the control group achieved 66% healing, the PBM group achieved 77.7% healing, and the HVPC group achieved 80% healing. The authors did not find any differences between the three intervention groups (*p* = 0.087), with no significant differences for abnormal protective sensations between the PBM and the HVPC group (*p* > 0.52). The authors concluded that there were no additional effects of PBM or HVPC to the standard wound care on healing of DFUs. The study did not mention race nor skin color of the participants.

de Carvalho et al. [36] conducted a clinical case study to evaluate the effects of PBM alone and associated with *Calendula officinalis* oil in treating DFUs. This was an experimental, randomized, controlled, and prospective interventional clinical case study that involved 32 patients of both genders with type 2 DM and aged 40–70 years old. Ulcers were located on the foot or in the medial or distal third of the leg and were between 1 and 5 cm in size. This study was designed to detect pain, wound size, ankle-brachial index (ABI), and Doppler readings. The participants were divided into four groups: group 1 was the control and received conventional standard treatment, group 2 received PBM (658 nm), group 3 received essential fatty acids (EFA; *C. officinalis*), and group 4 received PBM together with EFA. The results showed no significant difference within the groups with regards to the ABI and Doppler readings; the data remained consistent till the end of the study. The PBM group (group 2) and the PBM together with essential oils (group 4) reported a significant reduction in both wound area and pain, showing that PBM has analgesic effects [36]. Since there was no reported significance in group 3 in wound size or pain, it can be assumed that the results observed in group 4 are largely due to PBM. The skin color nor race of the participants was not mentioned in this study.

Mathur et al., [37] investigated the efficacy of PBM for the treatment of DFUs in a tertiary care center. This randomized controlled trial included a total of 30 patients with type 2 DM that presented with Wagner grade I foot ulcers for more than 6 weeks. The control group consisting of 15 patients who were treated with conventional treatment only. The study group was treated with PBM (wavelength 660 ± 20 nm) along with conventional therapy. The results showed that the PBM group had a significant reduction in wound size just after 2 weeks compared to the control group. The reduction in ulcer area was 37 ± 9% (30-50% wound area reduction observed in 75% of patients) in the PBM group compared to 15 ± 5.4% in the control group (80% of wounds showed a wound area reduction of <20%). The study group also showed increased granulation tissue. The results show significant benefits in patients treated with PBM over patients not treated with PBM. No adverse events and side effects were reported [36]. Skin color nor race of participants was mentioned in this study, though from a photograph provided in the article some participants may have had dark skin tones; however, this is based on assumptions and not facts provided for in the article.

A double-blinded, randomized study was conducted by Frangež et al. [38] on 60 patients which examined the influence of PBM with LEDs on chronic diabetic wound healing. Patients were randomized into an active (LED, 625 nm, 660 nm, and 850 nm) group (30 patients) or a control (placebo) group (30 patients). Results showed that after both the fourth and eighth week, the LED group had a remarkable improvement in wound bed granulation as well as a reduction in fibrin and eschar. After eight weeks, the mean wound surface was 56% of the starting wound surface in the LED group and 65% in the control group; there was a slight, although insignificant, faster reduction in wound surface area in the LED group. The patients in this study did not experience any side effects [38]. The race nor skin color of the participants was mentioned in this study.

Tantawy et al. [39] conducted a randomized controlled trial comparing the use of PBM at two different wavelengths in the management of DFUs. The clinical trial took place at the outpatient clinic of the Faculty of Physical Science at Cairo University for three months. Sixty-five participants were enrolled with ages ranging from 45 to 60 years, and with DFUs classified as grade I and II (Wagner Scale). Participants were randomly assigned to two groups; group I (33 participants) received PBM using a helium-neon laser and conventional wound care and group II (32 participants) received PBM at a wavelength of 904 nm and conventional care. Ulcer area was calculated from impressions on cellophane paper to graph paper. The two groups had similar baseline characteristics; however, after 4 and 8 weeks, there was a significant reduction in ulcer size (surface area) in both groups, with no significant differences between the two groups. The researchers did not mention the race nor participant skin color.

The study by Priyadarshini et al. [40] was a randomized controlled trial that consisted of 100 patients with Wagner grade I and II DFUs. The control group was treated with conventional therapy only, while the study group was treated with PBM (wavelength 660 nm) daily for 15 days. The ulcer size, grade, and culture status were assessed on day 1 and day 15. After 15 days, the study group showed improvement and healing; the ulcer size reduced and so was the grade as compared to the control group. The mean wound area at the start of the study in the control group was 19.09 ± 15.03 and 13.74 ± 11.88 in the PBM group. After treatment for 15 days, the mean wound area in the control group was 18.80 ± 17.70 compared to 3.97 ± 5.41 in the PBM group. At the start of the study, on day 1, 29 patients had grade II ulcers and 21 patients had grade I ulcers in the PBM group. At the end of the study, on day 15, there were no grade II ulcers; of the 29 grade II ulcers, 28 (96.6%) improved to grade I and one ulcer was completely healed. Of the 21 grade I ulcers, seven (33.33%) remained in grade I and 14 (66.67%) were completely healed. At the start of the study, on day 1 in the control group, 26 ulcers were classified as grade II and 24 as grade I. At the end of the study, on day 15, 23 (88.46%) grade II ulcers remained, and three ulcers (11.53%) improved to grade I. None of the grade I ulcers healed completely, and they all remained in grade I. In the PBM group, 31 patients had bacterial growth on day 1, of which 10 still had positive bacterial cultures at the end of 15 days. In the control group, 34 patients had bacterial growth on day 1, of which 29 still had positive bacterial cultures at the end of 15 days. This study concluded that PBM is a painless, cost-effective procedure that accelerates wound healing. No adverse events and side effects were reported [40]. Skin color nor race of participants was mentioned, though from a photograph provided in the article some participants may have had dark skin tones; however, this is based on assumptions and not facts provided for in the article.

de Alencar Fonseca Santos et al. [41] conducted a clinical trial to evaluate the effect of PBM on tissue repair processes in chronic DFUs. This was a randomized clinical trial with qualitative and quantitative prospective experimental methods. The clinical trial was carried out on 18 patients, with noninfected chronic DFUs. The patients were randomly divided into two groups: the control and the PBM group. The control group was treated with conventional treatment and the PBM group was treated with conventional treatment and PBM (660 nm). The pressure ulcer scale (PUSH) for healing was used to observe the tissue repair process weekly and it looked at the area of the wound, the exudate quantity, and the appearance of the wound bed. Higher PUSH scores indicated no ulcer improvement and lower PUSH scores indicated improvement in the healing of the wound. Pain intensity was analysed weekly using the visual analogue scale (VAS). de Alencar Fonseca Santos et al. [41] noted that the PBM group had a significant increase in tissue repair index as compared with the control group. The average final area in the control group was 1.63 cm^2^, while the average final area in the PBM group was 0.32cm^2^. There were no differences in the PUSH scale. It was concluded that the PBM group had an effective response in the tissue repair process in a short period of time. The skin color nor race of participants was not mentioned in this study.

Vitoriano et al. [42] conducted a prospective comparative clinical trial on laser and LED influence on tissue repair and improvement of neuropathic symptoms during the treatment of diabetic ulcers. This study consisted of twelve patients who were randomized in two groups; one group was treated with a GaAIAs laser (wavelength 830 nm) and the other group was treated with LED (wavelength 850 nm). The study took place over a period of 6 months, with sessions twice a week and patients were observed at the end of the 10^th^ session. Both groups showed a significant reduction in wound size at the end of treatments as compared to the start of the study. The results showed that the laser group had 81% wound reduction size by the 10^th^ session as compared to the LED group which had 62% wound size reduction. Although participants in the laser group had a significant wound size reduction as compared to the LED group, both treatments resulted in a decreased score of neuropathic signs and symptoms. There were no side effects noted in this study [40]. The skin color nor race of participants was not mentioned.

A clinical case study was done by de Alencar Fonseca Santos et al. [41] on self-administered PBM on diabetic leg ulcers. This case study was performed on an 84-year-old Chinese woman who had type 2 DM for 32 years and presented with diabetic complications such as neuropathy, vascular disease, eye disease, and leg ulcers. The leg ulcers had been present for 3 weeks and the biggest leg ulcer had a diameter of 1 cm. The B-cure laser pro device (808 nm) was used by the patient at home and was applied twice daily for 15 min. The results showed that after a week of treatment the smallest ulcer dried and crusted, while the larger ulcer significantly improved. All ulcers were completely healed after 30 days of treatment. There were no side effects mentioned in this case study [41]. The study supported the home use of PBM for the self-treatment of DFUs.

## 4. Conclusion

Conventional therapy alongside the use of PBM has shown significant efficacy as treatment modalities for diabetic ulcers. PBM seems to be an efficient, viable, painless method of treating DFUs. The available studies on the use of PBM for diabetic wounds encourage further investigation. There are not enough properly designed, randomized, placebo-controlled clinical trials on a larger group to determine the safety and efficacy of PBM, especially taking skin color into consideration. More randomized clinical trials with variable doses and treatment protocols for different wounds on patients of a specific, darker skin color (or animal studies) should be carried out so as to determine if skin color does affect PBM treatments. However, the studies that have been published show great promise for this therapy. Most studies conducted thus far on PBM do not mention the skin color nor race of patients, which should be considered due to varying photon absorbencies by melanin. Only one of the studies evaluated in this review took skin color into consideration and adjusted the dose accordingly. However, despite these potential shortfalls, all the studies showed an improvement in the healing rate of DFUs treated with either red or NIR light. More studies on the healing of DFUs with PBM must be done and these studies should focus on the skin types/colors.

## Figures and Tables

**Table 1:** Clinical studies and trials on diabetic ulcers treated with photobiomodulation (PBM).

Author	Study design	Participants	Interventions	Results
Kazemi-Khoo [29]	Case study	Seven diabetic patients (4 males and 3 females) with grade II and III diabetic ulcers.	PBM of ulcer beds (wavelength 660 nm; power 25 mW; fluence 0.6-1.0 J/cm^2^) and wound margins (wavelength 980 nm; power 200 mW; fluence 4-6 J/cm^2^) for 15–20 min; intravenous PBM (wavelength 655 nm; power 2 mW; irradiation time 20 min); and laser acupuncture with infrared light (wavelength not provided; 1 J/cm^2^) for LI-11, LI-6, SP-6, PC-6, ST-36, and GB-34 points. PBM sessions were every other day for 10–15 sessions (route 1) and then twice weekly (route 2) until complete recovery was achieved.	Complete wound closure in an average of 19 sessions, with no reported side-effects and no ulcer recurrence at 6 months. The race nor skin color of the participants was not mentioned; however, images used indicate light-skinned patients were used for the trial.
Saltmarche [30]	Prospective comparative clinical trial	Sixteen residents (9 females and 7 males) at a nursing home with 21 open wounds, including pressure ulcers, venous ulcers, and diabetic wounds.	Two 80 mW laser clusters with wavelength 785 nm (16 x 5 mW) were used to treat the wound periphery and wound bed. A 50 mW laser probe of the same wavelength treated deeper tissue. Wound margin received a fluence of 2–4 joules (1 min), the wound bed received 1–2 joules (30 seconds), and the eschar received 4–6 joules (2 min). For patients with darker skin tones, the dose around the periphery was increased by 50%. PBM administered 5 times for the first week and 3 times a week for weeks 2 to 9 or until healed. PBM was combined with the Extendicare Wound Prevention and Management Program.	61.9% of open wounds had a reduction in size (>50% wound closure) and 42.8% had complete closure; 23.8% of wounds demonstrated no change. No significant differences between chronic and acute wounds were evident. Skin color nor race of participants was mentioned.
Minatel et al., [31]	Double-blinded randomized placebo-controlled clinical trial	Fourteen patients with a total of 23 diabetic leg ulcers. Group one had 10 ulcers and group two had 13 ulcers. The gender of patients was not mentioned.	All patients received conventional therapy (cleaned and dressed with 1% silver sulfadiazine cream, covered with gauze and bandage) twice a week. Group one received placebo PBM of <1 J/cm^2^ from a probe with three disabled 660 nm diodes and a resistor added to a fourth diode (<5 mW average power and<1 mW/cm^2^ irradiance). Group two was treated for 30 s/spot with PBM using a 5 cm^2^ probe cluster with 36 diodes (32 diodes at 890 nm and 4 diodes at 660 nm) of average power of 500 mW (100 mW/cm^2^) and fluence of 3 J/cm^2^.	Ulcers in group one healed rapidly within the first 30 days, while ulcers in group two deteriorated, with 56% more granulation and 72% faster healing rate in group one. By day 90, 58.3% of group two had fully healed ulcers and only one ulcer was fully healed in group 2. Group two ulcers achieved a 90–100% healing, while only a single ulcer attained >90% healing in group two. No side effects were experienced. Skin color nor race of participants was mentioned.
Kaviani et al., [32]	Double-blinded, randomized, placebo-controlled clinical trial	Twenty-three diabetic patients (13 in the PBM group and 10 in the placebo group) with ulcers stage I and II (Wagner scale) with a mean duration of 10 months.	PBM (wavelength 685 nm; fluence 10 J/cm^2^; density 50 mW/cm^2^) for 200 s 6 times per week for the first two weeks, then every other day till complete healing. Noncontact mode was used at a distance of 1 cm. All patients received conventional therapy.	The wounds reduced in size and healing time in the PBM study group compared to the placebo group. Ulcers in the PBM group achieved 66.6% complete healing compared to 38.4% in the placebo group. Neither race nor skin color of the participants was mentioned.
Kajagar et al., [33]	Randomized-controlled clinical trial	Sixty-eight patients (male:female ratio 3 : 1) with type 2 DM with Wagner grade I foot ulcers (34 patients as controls and 34 patients as the study group).	Treatments were performed daily for 15 days (wavelengths not provided; power 60 mW; fluence 2-4 J/cm^2^; pulse frequency 5 kHz). Only the wound bed and wound edges were irradiated then covered with conventional moist dressing. Offloading took place in patients with plantar pressure.	Significant reduction in ulcer size in the PBM group was noted. The mean reduction in ulcer size was 1043.20 ± 266.62 mm in the PBM-treated group compared to 322.44 ± 85.84 mm in the control group. The researchers did not mention participant's skin color/race.
Feitosa et al., [34]	Randomized-controlled clinical trial	Sixteen patients with type 2 DM (8 patients as control and 8 patients as the study group).	PBM (wavelength 632.8 nm; power 30 mW; fluence 4 J/cm^2^; irradiation time 80 s) administered for 12 sessions of which 3 were weekly sessions, on alternated days. Pain evaluation after 30 days follow-up. Both groups had the wounds washed with 0.9% saline solution.	Decrease in wound size in study group as compared to the control group. Intense improvement in pain in study group. The average wound area (cm^2^) before treatments in the control group was 2.55 ± 0.77, which increased to 8.43 ± 1.84 after 30 days. In the study group, a significant decrease in wound size was noted and patients reported an improvement in pain. The average wound area (cm^2^) before treatments in the PBM group was 7.98 ± 2.06, which decreased to 2.39 ± 1.26 after 30 days. The race nor skin color of the participants was not mentioned.
Ortíz et al., [35]	Randomized-controlled clinical trial	Twenty-eight patients with DM and ulcers on the distal legs or feet were randomized into 3 groups (control group, PBM group, and high-voltage-pulsed current group, HVPC).	Groups were treated for 16 weeks. The control group received standard care and wounds were irrigated with saline and received debridement and application of wound dressings 7 days a week. The HVPC group was treated for 45 min three times a week with an electrical simulator (pulse of 100 pps, 100 *μ*s pulse duration) with standard wound care protocol. The PBM group was treated three times a week at a wavelength of 685 nm, (30 mW, applied at 2 J/cm^2^ (18 s) on the wound edges in light contact, and 1.5 J/cm^2^ (14 s) on the wound bed in noncontact mode).	The control group achieved 66% healing, the PBM group achieved 77.7% healing, and the HVPC group achieved 80% healing. There were no significant differences between the three groups (*p* = 0.087). No significant differences were found for protective sensations between the PBM and the HVPC group (*p* > 0.52). The race nor skin color of the participants was not mentioned.
de Carvalho et al., [36]	Experimental, randomized, controlled, prospective, and interventional clinical case study	Thirty-two patients with type 2 DM and foot and leg ulcers randomized into 4 groups (group 1 control, group 2 PBM group, group 3 essential oil group, and group 4 PBM with essential oil group).	Control group treated daily with conservative treatment (wound cleaned with 0.9% saline and covered with sterile gauze) for 30 days. PBM group treated with conservative therapy and PBM (wavelength 658 nm, power 30 mW, and fluence of 4 J/cm^2^) using contact mode at equidistant points around and on the wound bed for 80 s, three times a week for a month. Group 3 was treated daily with conservative therapy and 5 mL calendula oil for 30 days. Group 4 was treated with conservative therapy, PBM as per group 2 and calendula oil as per group 3 for 30 days.	No significant changes in ankle-brachial index (ABI) and Doppler readings from onset to end of the study in all groups. Reduction in wound size and pain noted in PBM group (*p* = 0.0428 and *p* < 0.001, respectively) and in PBM with essential oil group (*p* = 0.0032 and *p* < 0.001, respectively). Neither race nor skin color of the participants was mentioned.
Mathur et al., [37]	Randomized controlled trial	Thirty patients (male:female ratio 2 : 1) with type 2 DM and Wagner grade I foot ulcers (15 patients as control and 15 patients as the study group).	PBM (wavelength 660 ± 20 nm; power density 50 mW/cm^2^; fluence 3 J/cm^2^; irradiation time 60 s) or conventional therapy alone. Daily PBM treatment for 15 days. Control group only treated with conventional therapy including daily wet saline or betadine dressings, antibiotic treatment, contact cast immobilization, and slough excision as and when required. Pressure offloading was carried out in patients with plantar ulcers.	The reduction in ulcer area was 37 ± 9% (30-50% wound area reduction observed in 75% of patients) in the PBM group compared to 15 ± 5.4% in the control group (80% of wounds showed a wound area reduction of <20%). The study group also showed increased granulation tissue. The results show significant benefits in patients treated with PBM over patients not treated with PBM. No adverse events and side effects were reported. Skin color nor race of participants was mentioned.
Frangež et al., [38]	Double-blinded, randomized, placebo-controlled clinical trial	Sixty patients with chronic diabetic ulcers (30 patients in the active group and 30 patients in the control group).	The active group was treated with pulsed wave-modulated LED (wavelength 625 nm, 660 nm and 850 nm; fluence 2.4 J/cm^2^) for 5 min. The control group received placebo LED (broadband wavelength of 580 nm to 900 nm; fluence 0.72 J/cm^2^) for 5 min. Patients were treated three times a week for 8 weeks at a distance of 10 cm from the wound. Both groups received conventional therapy (debridement, moist wound bed, and infection control).	Improvement in wound bed granulation and reduced fibrin and eschar. Faster (insignificant) healing rate observed in PBM group. Race nor skin color of the participates was mentioned.
Tantawy et al., [39]	Randomized-controlled clinical trial	Sixty-five patients (51 males and 14 females) aged 45-60 years and with DFUs classified as grade I and II (Wagner scale).	Patients randomly assigned to two groups; group I received PBM using a helium-neon laser (632 nm, peak power of 20 mW, pulse frequency of 25 Hz, and power density of 15 mW/cm^2^, surface area with 90 s application/cm^2^, and dose of 5 J/cm^2^) and conventional wound care (dressings with wet betadine or saline, antibiotics, and debridement if necessary). Group II received PBM at a wavelength of 904 nm (peak power of 20 mW, pulse frequency of 25 Hz, power density of 40 mW/cm^2^, spot size of 1 cm^2^; time of application was determined according to the surface area with 90 s/cm^2^ and dose of 6 J/cm^2^) and conventional care.	There was a significant reduction in ulcer size in both groups at 4 and 8 weeks with no significant differences between the groups. Group I had a baseline ulcer size (cm^2^) of 10.2 ± 5.6, with ulcer sizes of 7.1 ± 1.5 and 3.7 ± 1.2 after 4 and 8 weeks, respectively, while group II had a baseline ulcer size (cm^2^) of 9.5 ± 4.2, with ulcer sizes of 7.8 ± 1.6 and 4.1 ± 1.3 after 4 and 8 weeks, respectively. Race nor skin color of the participates was mentioned.
Priyadarshini et al., [40]	Randomized controlled trial	One hundred patients (31 males and 19 females in control; 26 males and 24 females in study group) with diabetic foot ulcers (50 patients as control and 50 as the study group) and Wagner grade I and II foot ulcers.	PBM (wavelength 660 nm; fluence 4-8 J/cm^2^; irradiation time 20 min) daily for 15 days. Control group treated with conventional treatment only including dressings with betadine or wet with saline, antibiotic treatment and slough removed when necessary.	Reduction in mean area of ulcers at day 15 was noted in the PBM group compared to the control group. There was 66% complete wound healing in participants with grade I ulcers and 96.6% grade II ulcers improved to grade I. The race nor skin color of the participants was not mentioned.
de Alencar Fonseca Santos et al., [41]	Randomized clinical trial	Eighteen patients aged 30–59 years old, with chronic noninfected diabetic foot ulcers, randomized into two groups (control group and the laser group).	Control group was treated with conventional treatment (cleansed wound bed with 0.9% physiological solution and 2 mg hydrogel, covered with gauze and bandage) every 2 days for four weeks. Laser group treated with conventional therapy and PBM (660 nm, power density of 30 mW, and fluence of 6 J/cm^2^) every 2 days for 4 weeks.	The PBM-treated group had a significant increase in tissue repair index. Average final area in the control group was 1.63cm^2^ compared with 0.32 cm^2^ in the PBM group. The skin color nor race of participants was mentioned.
Vitoriano et al., [42]	Prospective, randomized comparative clinical study	Twelve participants with chronic diabetic ulcers randomized into two groups (the laser group and the LED group).	Laser group treated with a GaAIAs laser (wavelength 830 nm; power 30 mW; density 0.25 W/cm^2^; fluence 7 J/cm^2^; contact mode). LED group treated with LED (wavelength 850 nm; power 48 mW; density 1.05 W/cm^2^; fluence 4.49 J/cm^2^, contact mode). The study took place over 6 months, with sessions twice a week and patients were observed at the end of the 10^th^ session.	Drastic wound reduction size in laser treated group as compared to the LED group; 81% wound reduction compared to 62%. Both groups displaced a decrease in neuropathic symptom scores. The race nor skin color of the participants was not mentioned.
Merigo et al., [43]	Case study	An 84-year-old Chinese female patient with type 2 DM and diabetic leg ulcers present for three weeks.	B-cure laser pro and conventional treatment were in place. PBM was self-administered at home. The device emitted infrared light at 808 nm (power output 250 mW; surface area 4.5 cm^2^; power density 55.5 mW/cm^2^; frequency 15 kHz; energy 14.4 J per minute; fluence 3.2 J/cm^2^ per minute; total fluence 48 J/cm^2^). The treatment was administered daily in two sessions for 15 min.	After one week of treatment the smallest ulcer dried while the larger ulcer was completely healed after thirty days.

## Data Availability

All articles used in this review are available from the respective journals concerned.
